# Geological implication of grain-size segregation in dense granular matter

**DOI:** 10.1098/rsta.2017.0390

**Published:** 2018-11-26

**Authors:** Ryo Itoh, Takahiro Hatano

**Affiliations:** 1 Dia Consultants Co. Ltd., 2-272-3 Yoshinocho, Kita-ku, Saitama 331-0811, Japan; 2 Earthquake Research Institute, University of Tokyo, 1-1-1 Yayoi, Bunkyo, Tokyo 113-0032, Japan

**Keywords:** fault gouge, grain size segregation, friction

## Abstract

To the current common belief, grain size segregation in granular matter requires sufficient porosity. Therefore, grain size segregation found in a natural fault gouge could imply elevated fluid pressure and the reduced normal stress on fault, possibly caused by the frictional heat during an earthquake. To clarify whether fluidization is essential to grain size segregation, we conduct numerical simulation on a simple model of fault gouge in a plane shear geometry under constant volume condition: the volume fraction is fixed at 0.6, at which the granular system possesses yield stress. We observe apparent grain size segregation at this volume fraction, meaning that grain size segregation alone does not imply fluidization of granular matter. We also show that segregation is driven by the nonlinear velocity profile, and that the gravity is not essential to segregation. The physical condition tested here may be relevant to earthquake faults: the normal stress of 1 MPa, the sliding velocity of 1 m s^−1^, and the duration of 0.1 s.

This article is part of the theme issue ‘Statistical physics of fracture and earthquakes’.

## 1. Introduction

A fault zone generally contains narrow slip bands consisting of fine rock powders that are referred to as fault gouge. The structures and textures of fault gouge are believed to provide geologists with rich information on the coseismic slip dynamics of faults. In other words, by observing the textures, we may infer physical processes that occurred in a past earthquake.

Monzawa & Otsuki [1] investigated rock samples taken from four fault zones in Japan and found some evidence for *gouge fluidization* that may occur during coseismic fault slip. Here fluidization is defined as the state in which grains have a non-zero free path like gaseous molecules and do not have enduring contact. Although this is apparently counterintuitive, some plausible mechanisms have been proposed, e.g. acoustic vibration [2] and thermal pressurization [3].

Irrespective of the mechanism behind, a fault can be much weaker if gouge fluidization occurs. Gouge fluidization is thus an elementary physical process of dynamic weakening of fault, which affects earthquake rupture propagation and strong ground motion. Therefore, it is important to verify the existence of gouge fluidization using various independent approaches: geological observation [4–6] and laboratory experiment [7,8].

There are some characteristic textures that are believed to be caused by gouge fluidization [1], e.g. injection structures and the spatial distribution of the fragment counterparts. Among such textures, in this Letter we focus grain size segregation. Here the grain size segregation refers to a state in which larger gouge particles migrate towards the upper part of a slip band, and smaller particles towards the lower part. In geological observation, grain size segregation was found in the Chelungpu fault gouge [9], where the coseismic slip was accommodated in the 1999 Taiwan Chi-Chi earthquake. In laboratory experiment, grain size segregation was also observed in the water-saturated clay-rich fault gouge subjected to large displacement with a high slip velocity of 1.3 m s^−1^ under the normal stress of 0.6–2.0 MPa [7,8]. In these studies, grain size segregation is supposed to be the Brazil-nut effect [10,11], which requires gravity and the sufficient porosity in granular matter. Then, grain size segregation in fault gouge may imply sufficiently high porosity in fault gouge, and therefore can be evidence for gouge fluidization.

Importantly, however, grain size segregation may not always necessitate the gravity [12–14]. In particular, Fan & Hill [15,16] conduct a numerical simulation on a vertical chute flow at relatively high density (the average packing fraction of 0.6), and find that the direction of segregation is perpendicular to the gravitation. They conclude that it is due to the non-uniform gradient in the flow velocity. Therefore, we may argue that grain size segregation does not always require gravity and high porosity.

We wish to know whether such a segregation mechanism works or not in a simple shear geometry that is relevant to earthquake faults. To address this problem, we investigate grain size segregation in a simple model of fault gouge by means of numerical simulation. We show that segregation occurs irrespective of the gravitation in a non-fluidized system at a high density, and that segregation is driven by the nonlinear velocity profile. This implies that grain size segregation alone does not evidence gouge fluidization.

## 2. Model

### (a) Force model

In this study, a three-dimensional system is investigated. The grains are assumed to be spheres, and the interaction force is described by the discrete element method (DEM) [17]. Consider a grain *i* of radius *R*_*i*_ located at **r**_**i**_, the translational velocity **V**_**i**_ and the angular velocity ***Ω***_**i**_. This grain interacts with another grain *j* whenever they are in contact; i.e. |**r**_*ij*_| < *R*_*i*_ + *R*_*j*_, where **r**_*ij*_ = **r**_*i*_ − **r**_*j*_. The force acting on the two particles constitutes of two components, each of which is normal or transverse to **r**_*ij*_. Introducing the normal unit vector **n**_*ij*_ = **r**_*ij*_/|**r**_*ij*_|, the normal force **F**^*n*^_*ij*_ is given by 
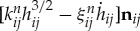
, where *h*_*ij*_ = *R*_*i*_ + *R*_*j*_ − |**r**_*ij*_|. The expression *k*^*n*^_*ij*_*h*^3/2^_*ij*_ is the normal elastic force described by Hertzian contact stress theory, where *k*^*n*^_*ij*_ is the normal elastic coefficient. The expression 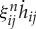 is the normal inelastic force, where *ξ*^*n*^_*ij*_ is the normal damping coefficient. These interaction parameters are expressed in terms of material parameters [18].
2.1
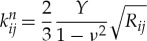
and
2.2
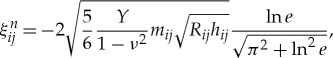
where *Y* is the Young modulus, *ν* the Poisson ratio, *e* the coefficient of restitution, *m*_*i*_ the mass of grain *i*, *m*_*ij*_ = *m*_*i*_*m*_*j*_/(*m*_*i*_ + *m*_*j*_), and *R*_*ij*_ = *R*_*i*_*R*_*j*_/(*R*_*i*_ + *R*_*j*_).

In order to define the transverse force, we define the relative tangential velocity as
2.3
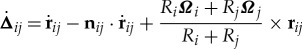
and introduce the relative tangential displacement vector **Δ**_*ij*_ as 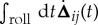. The subscript of the integral indicates that the integral is performed only when the contact is *rolling*; i.e. |*k*^*t*^_*ij*_**Δ**_*ij*_| < *μ*_*e*_|**F**^**n**^_**ij**_|, where *k*^*t*^_*ij*_ is the tangential elastic coefficient and *μ*_*e*_ the inter-particle friction coefficient. The tangential elastic coefficient and the tangential damping coefficient are expressed by the following equations [18]:
2.4
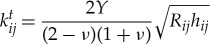
and
2.5
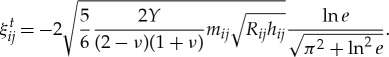
The magnitude of tangential force depends on the state of the contact: |*k*^*t*^_*ij*_**Δ**_*ij*_| for rolling contact, or *μ*_*e*_|**F**^**n**^_**ij**_| otherwise.

### (b) Configuration

There are two types of gouge grains in this simulation: small grains and large grains. The diameter of the large grains is 2*d*, whereas the diameters of small grains are uniformly distributed in [0.8*d*, 1.2*d*] to ensure disordered packing of the grains. Here *d* is the length constant and set to be 10 μm in this study. The numbers of small grains and large grains, respectively, are 1120 and 80. The grain mass is given by *ρπd*^3^/6, where *ρ* is the mass density.

The dimensions of the system are set to be *L*_*x*_ = 12*d*, 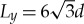 and *H* = 13.2*d*, where we use periodic boundary conditions in the *x*- and the *y*-directions. There exist two walls at the boundaries of the *z* direction. The walls consist of small grains (diameter *d*) aligned on the regular triangular lattice, except otherwise indicated. They interact with the bulk grains via the force described above. The lower wall is displaced along the *x*-axis at constant velocity *V* , whereas the upper wall is fixed. Here we set *V* = 1 m s^−1^ throughout this study. This realizes a plain shear, where the velocity gradient is formed in the *z*-direction. These walls are completely rigid and not allowed to move along the *y*- and *z*-axes. Namely, the volume is kept fixed in this study.

Here we address whether fluidization is essential to grain size segregation in granular matter. Because fluidization is controlled by volume fraction, it is more illustrative to work at constant volume condition at which granular matter is not fluidized. Here we focus on the volume fraction *ϕ* = 0.6, where the system has the yield stress [19]. The results for other densities will be presented elsewhere. The experimental parameters are tabulated in table 1. The time step in the simulation is Δ*t* = 5.0 × 10^−10^ s.

**Table 1.** Physical parameters used in the simulation.

parameters	value
end dimensional constants	
characteristic grain size *d*	10 μm
mass density *ρ*	2700 kg m^−3^
Young's modulus *Y*	55 GPa
non-dimensional constants	
Poisson's ratio *ν*	0.25
coefficient of restitution *e*	0.5
inter-particle friction coefficient *μ*_*e*_	0.5

### (c) Preparation of the initial system

**Figure 1.**
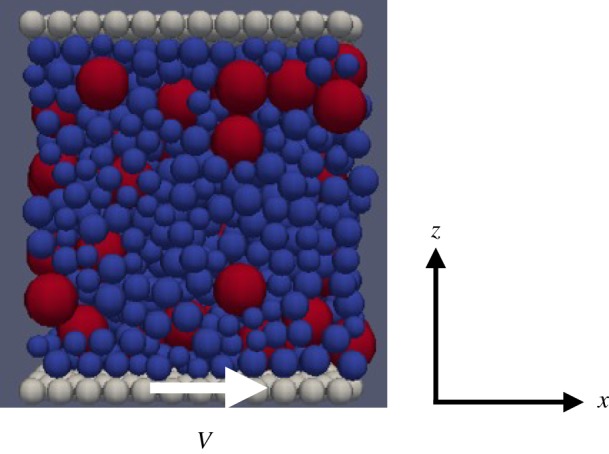
The system geometry of the initial state. The upper wall is kept fixed, while the lower wall is displaced at constant velocity, *V* . The *y*-axis is out of plane. (Online version in colour.)

Initially, the lower and the upper walls are set at *z* = 0 and *z* = 84*d*, respectively. Within this range, the gouge grains are distributed on the cubic lattice, the unit cell size of which is 2*d*. Each grain is given the velocity of 0.1 m s^−1^ in a random direction. At the same time, the upper wall is displaced downward along the *z*-axis at the velocity of 0.05 m s^−1^. In 0.011 s, the initial lattice configuration disappears due to the random velocity, and then the gravity is applied. After 0.035 s of relaxation under gravity, the maximum grain velocity decreases to the order of 10^−5^ m s^−1^. The movement of the upper wall continues until the width between the upper and the lower walls becomes 13.2*d*. This defines the initial state at *t* = 0. Then, at *t* = 0, the lower wall is displaced at constant velocity of 1 m s^−1^ in the *x*-direction. This causes the simple shear, and the velocity gradient is formed in the *z*-direction. Throughout the present simulation, the vertical positions (the *z*-coordinates) of the walls are fixed and therefore the volume of the system is constant. The geometry of the simulation system is shown in figure 1.

While the shear causes dilation under a constant pressure condition, the shear increases the normal stress under a constant volume condition. Thus, at a steady state, the normal stress fluctuates in time under a constant volume condition. The time-averaged normal stress at a steady state is approximately 1 MPa, and from this we can compute the inertial number of the present situation as *I*≃2.8 × 10^−3^ [20].

Because the detailed configuration of grains depends on the random velocity of 0.1 m s^−1^ given at the earliest stage, we can prepare many samples of different grain configuration using different seeds for random number. We confirmed that the overall tendency of the result does not depend on this randomness, and therefore we describe the result for one sample in the following unless otherwise indicated.

## 3. Results

To check the effect of gravity quantitatively, we investigate two cases: with gravity (*g* = 9.8 m s^−2^) and without gravity. The latter is realized simply by setting *g* = 0 at *t* = 0. In both cases, the normal stress on the walls is approximately 1 MPa at a steady state, while the normal stress in the zero gravity case is slightly (approx. 10%) smaller than that in the gravitational case. In the literature of earthquake faults, the system with gravity may be interpreted as dip slip, whereas the system without gravity may be interpreted as lateral slip. Hereafter we discuss the both cases in parallel.

### (a) Segregation tendency

**Figure 2.**
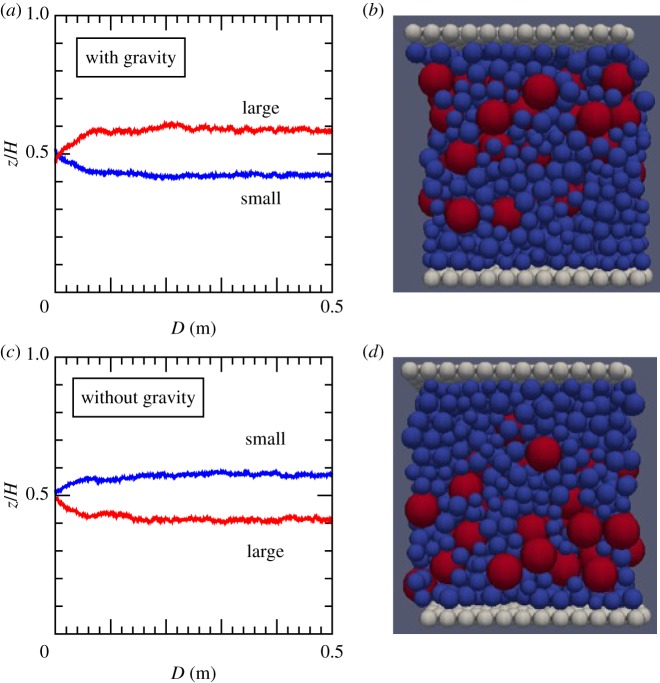
Evolution of the mean positions and the snapshots of the evolved states. (*a*,*c*) The average *z*-coordinates of the small (blue) and the large (red) grains normalized by the granular layer thickness, *H*, as a function of slip displacement. (*b*,*d*) The snapshots of the evolved states. (*a*,*b*) With gravity and (*c*,*d*) without gravity. (Online version in colour.)

First, we trace the centre of mass for each type of grains: large grains and small grains. Figure 2 shows the temporal evolution of the average *z*-coordinates of the small and the large grains, respectively. As they are normalized by the granular layer thickness *H*, the value of 0.5 means that the grains are uniformly distributed. The normalized *z*-coordinate is 0.5 at *t* = 0. Namely, the initial system is uniform. Then, as shown in figure 2, segregation begins as soon as the wall is displaced and reaches the steady state at the displacement of 10 cm. This is the characteristic slip distance for grain size segregation for the parameters adopted here. We find that segregation occurs irrespective of gravitation, and the characteristic slip distance for segregation does not seem to depend on gravitation. In both cases, the volume fraction is fixed at 0.6 and the normal stress is approximately 1 MPa. Note that this is the actual normal stress applied to grains, and therefore grain size segregation does not necessarily imply vanishing normal stress.

**Figure 3.**
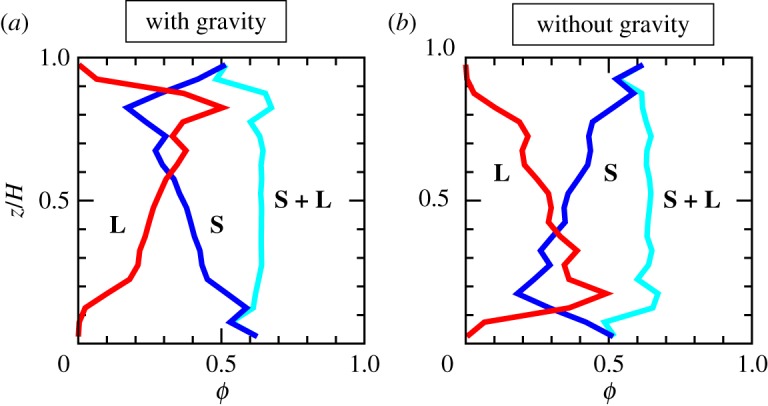
Volume fraction profile. (*a*,*b*) Volume fraction profiles of small grains (the blue line marked with **S**), of large grains (the red line marked with **L**), and of the all grains (the light-blue line marked with **S** + **L**). Each profile is averaged during the displacement of 40–50 cm. The horizontal axis is volume fraction *ϕ* and the vertical axis is the distance from the lower wall, *z*, normalized by the granular layer thickness, *H*. (*a*) With gravity and (*b*) without gravity. (Online version in colour.)

Figure 3 shows the density profiles of each type of grains at the final stage of simulation, when the slip displacement of the lower wall is 40–50 cm. The profiles are averaged during this slip. In the presence of gravity, large grains segregate upward (that is, opposite to the direction of gravity). This tendency is the same as the Brazil-nut effect. Importantly, however, segregation also occurs in the absence of gravity, and therefore there must be a common mechanism for segregation other than the gravity.

### (b) Velocity profile

**Figure 4.**
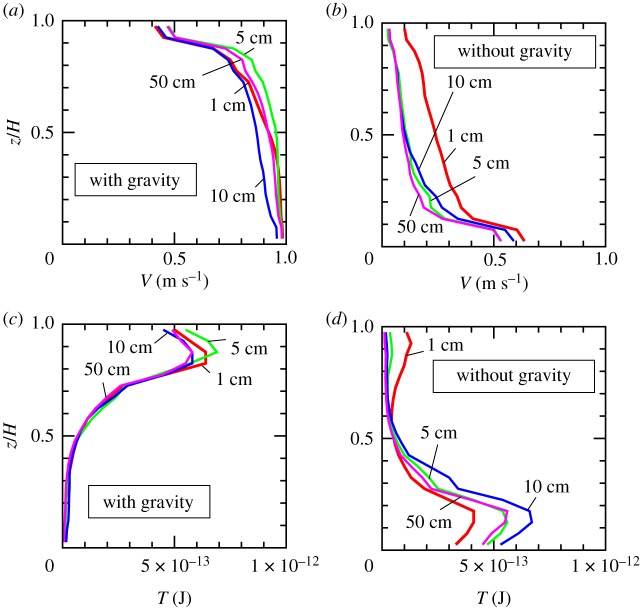
Velocity profile and temperature profile. (*a*,*b*) Velocity profiles averaged during the displacement of 0.5–1 cm (the red line), 4.5–5 cm (the light-green line), 9.5–10 cm (the blue line) and 49.5–50 cm (the pink line). The horizontal axis is the velocity in the *x*-direction and the vertical axis is the distance from the lower wall, *z*, normalized by the granular layer thickness, *H*. (*c*,*d*) Profiles of the granular temperature. See text for the definition. The colourings are the same as those in (*a*) and (*b*). (Online version in colour.)

In figure 4, the time evolution of the velocity profiles are shown. Irrespective of gravitation, the velocity profile reaches a steady state after the displacement of 10 cm. This takes approximately 0.1 s.

Note that the steady-state velocity profile is nonlinear. The nonlinear velocity profile appears to be steady within the simulation duration. In the presence of gravity, velocity gradient is larger in the upper part of the sample (figure 4*a*). This is opposite in the case without gravity (figure 4*b*).

Combining this result with the segregation tendency shown in figure 3, and also considering the result reported in [15,16], one may conclude that large grains segregate in the region of large velocity gradient. Namely, size segregation here is caused by the nonlinearity in the velocity profile, but not by the gravity.

Because the velocity fluctuation is an increasing function of velocity gradient [14,20], velocity fluctuation may be large in the region of large velocity gradient. Thus, we may also interpret that inhomogeneity in the velocity fluctuation, referred to as *granular temperature*, causes segregation. In figure 4*c*,*d*, the profiles of granular temperature are shown. Here granular temperature is defined by
3.1
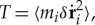
where 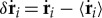. The average 〈 · 〉 is defined in each local layer of *z* = const. plane, and taken in a certain duration. In the view of figure 4, one can conclude that large grains segregate towards a region of high velocity fluctuation.

**Figure 5.**
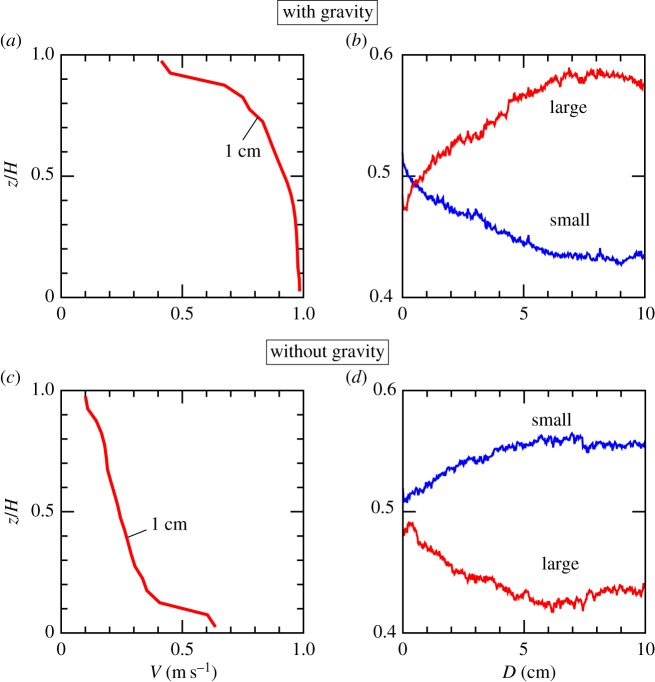
Velocity profile and the temporal segregation tendency. (*a*,*b*) Simulation with gravity. (*a*) Velocity profile averaged during the displacement *D* = 0.5–1 cm. The horizontal axis is the velocity in the *x*-direction and the vertical axis is the distance from the lower wall, *z*, normalized by the granular layer thickness, *H*. (*b*) The average *z*-coordinates of the small (blue) and the large (red) grains normalized by the granular layer thickness, *H*, as a function of slip displacement. (*c*,*d*) Simulation without gravity. (*c*) Velocity profile averaged during the displacement *D* = 0.5–1 cm, when the fault slips without gravity. The horizontal axis is the velocity in the *x*-direction and the vertical axis is the distance from the lower wall, *z*, normalized by the granular layer thickness, *H*. (*d*) The average *z*-coordinates of the small (blue) and the large (red) grains normalized by the granular layer thickness, *H*, as a function of the slip displacement. (Online version in colour.)

One may argue that the nonlinear velocity profile (i.e. inhomogeneous granular temperature) may be rather a result of grain size segregation, but not the cause. To reject this argument, we show that the nonlinear velocity profile is formed before segregation. Figure 5 shows the transient process of the segregation before the system reaches the steady state. Figure 5*a*,*b* are for the case of gravitation. In figure 5*a*, the nonlinear velocity profile is apparent at the displacement of 1 cm, whereas the segregation just started and the system does not reach the steady state at this instance (figure 5*b*). This is also true in the case without gravity as shown in figure 5*c*,*d*. Namely, the nonlinear velocity profile is formed before segregation. Thus, the argument that segregation causes the nonlinear velocity profile is dispelled.

### (c) Cause of the nonlinear velocity profile

In principle, the linear velocity profile is expected in a simple shear geometry and thus the nonlinear velocity profile obtained here may be unexpected. Nevertheless, similar nonlinear velocity profiles are observed in a wide class of particulate systems under the simple shear [21–25]. In this sense, the nonlinear velocity profile in the simple shear is not very surprising. But a general theory still does not exist despite some promising attempts [23,26,27]. Hereafter we discuss some potential origins of the nonlinear velocity profile.

#### (i) Effect of initial packing

To test the robustness of the nonlinear velocity profiles, we conduct simulations on many samples of different random configurations. Namely, each sample is given different initial random velocities at the preparation stage. (See §2c.) As a result, grain packing at *t* = 0 is different from sample to sample. We test 10 samples and, importantly, obtain nonlinear velocity profiles in all the runs. Therefore, the nonlinear velocity profile is robust irrespective of the detailed configuration of the grains.

In each simulation of the 10 independent samples, the sense of convexity of the velocity profile is different from sample to sample. It has the same shape as in figure 4 in five samples, while it is opposite in the other five cases: it appears to be random. Therefore, the sense of convexity of the velocity profile may be determined by the randomness in grain packing. Importantly, however, the segregation tendency in each sample always corresponds to the shape of the velocity profile: large grains segregate toward the region of large velocity gradient. This also supports our claim that the nonlinear velocity profile drives grain size segregation.

One may still suspect that the initial packing has some asymmetry: e.g. The upper part of the system could be of lower density because the initial packing is prepared by gravitation. However, the volume fraction of the initial sediment is lower than 0.6 and it must be compressed by the upper wall to reach the volume fraction of 0.6. As a result, the initial volume fraction becomes approximately uniform with respect to the depth.

#### (ii) Effect of the wall acceleration

As the wall velocity goes from *V* = 0 to 1 m s^−1^ at *t* = 0 in one time step, one may suspect that the large wall acceleration causes artificially large forces on the grains and leads to the nonlinear velocity profile. To check this possibility, we perform additional simulations in which the wall motion is ramped gradually. The wall is accelerated from 0 to 1 m s^−1^ in 0.05 s, while the entire duration of the simulation is 0.15 s. The result is unchanged: the velocity profile becomes nonlinear and large grains segregate to the region of large velocity gradient. We thus conclude that the nonlinear velocity profile is not due to the stepwise change of the wall velocity.

#### (iii) Effect of wall roughness

**Figure 6.**
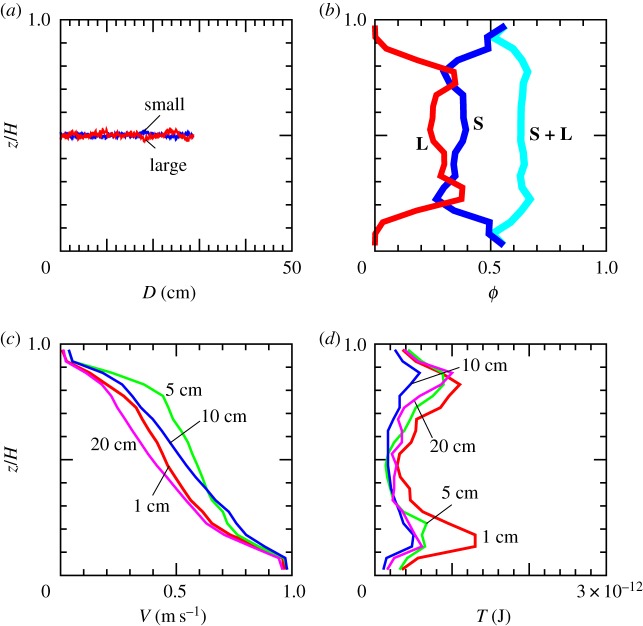
Simulation results for the rough walls. (See text for the details of simulation.) Gravity is not applied. (*a*) The average *z*-coordinates of the small (blue) and the large (red) grains normalized by the granular layer thickness, *H*, as a function of slip displacement. (*b*) Volume fraction profiles of small grains (the blue line marked with **S**), of large grains (the red line marked with **L**), and of the all grains (the light-blue line marked with **S** + **L**). Each profile is averaged during the displacement of 19.5–20.0 cm. (*c*) Velocity profiles taken at each displacement (indicated in the panel). (*d*) Granular temperature profiles taken at each displacement (indicated in the panel). (Online version in colour.)

Next we check the effect of the roughness of the walls. In the above simulations, the walls consist of grains of diameter *d* located on a triangular lattice. Then, to increase the roughness of the walls, we add grains of the same size on the second layer, which is separated from the first layer by 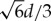. The second layer constitutes a rectangular lattice, the unit lattice of which is 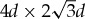. Owing to the existence of the sparse second layer, the walls may be regarded as more rough. We perform the simulation with the new walls without gravity. We obtain the linear velocity profile as shown in figure 6*c*, and, importantly, segregation does not occur as shown in figure 6*a*,*b*. Therefore, we can conclude that the nonlinear velocity profile is vital to grain size segregation in a simple shear geometry.

## 4. Discussions and conclusion

### (a) Physical cause of segregation

In the present simulation, we confirm the nonlinear velocity profile and the inhomogeneous granular temperature profile in granular matter under simple shear. Importantly, grain size segregation occurs at volume fraction of 0.6, where the system possesses yield stress, namely the system is not fluidized. Therefore, we can conclude that gouge fluidization is not essential to grain size segregation, and therefore grain size segregation does not necessarily imply fluidization.

We also find that large grains segregate in the region of large velocity gradient, or equivalently, of large granular temperature. This tendency of segregation is irrespective of gravitation. We confirm that the nonlinear velocity profile is formed before segregation, and that segregation does not occur if the linear velocity profile is realized. From these numerical results, we can conclude that the nonlinear velocity profile drives grain size segregation. This conclusion is consistent with some previous studies on other flow geometries [12,13,15,16], where large grains segregate toward the region of large velocity gradient.

Because the linear velocity profile is formed when we use a double-layered rough wall, the boundary effect may play a vital role to the shape of the velocity profile. This could be explained by a non-local constitutive law [27] in the presence of gravity, but it does not apply to the nonlinear profile formed under zero gravity. Thus, the reason for the nonlinear velocity profile in a simple shear geometry is not very clear at this point.

It is also important to know why large grains segregate towards the region of large velocity gradient (equivalently, the region of large granular temperature). It is well known that temperature gradient yields mass flow. In the context of granular matter, using a kinetic theory, Jenkins & Mancini show that granular temperature gradient drives segregation and predict that small grains migrate toward higher temperature region [28]. This is opposite to our result. As our system is of high density at which yield stress exists, it is not surprising that a kinetic theory does not apply. A quantitative theory does not exist that applies to granular systems of higher densities, but a heuristic and qualitative argument is as follows. Because the diffusion coefficient is an increasing function of granular temperature, grains tend to move from a region of higher granular temperature to that of lower granular temperature. As small grains are easier to pass between grains than large ones, small grains migrate toward lower granular temperature region eventually, and large grains are left behind in higher granular temperature region. A quantitative argument may trace a theory of thermophoresis (i.e. the Soret effect). Additionally, dependence of the segregation rate on the local relative density is an important ingredient that controls the mechanism of segregation [29].

One may wonder if grain-size segregation here is also caused by vibration-induced convection. However, the convection does not exist in our system and the mean flow is just the plain (but nonlinear) shear. In our system, inhomogeneous granular temperature is caused by the nonlinear flow velocity profile, and this is the cause of grain-size segregation. However, at this moment, we cannot conclude whether both ingredients are essential or inhomogeneous granular temperature alone can cause segregation. To check if the inhomogeneous granular temperature alone can case segregation, one should come up with a geometry in which inhomogeneous granular temperature is realized without the shear. (This might be realized by tapping.) In such geometries, granular convection may be formed spontaneously, probably as a result of inhomogeneous granular temperature. Thus, the flow and the inhomogeneous granular temperature can hardly be separated. A general relationship between the flow and the inhomogeneous granular temperature is a very interesting topic, but it is beyond the scope of this short paper.

### (b) Implication to earthquake faults

In this study, the fault gouge layers in a natural fault are modelled as granular matter subject to plain shear. Although the model is simplified, some of the conditions adopted in the present simulation may be relevant to natural earthquake faults. The slip velocity of 1 m s^−1^ is a typical maximum slip velocity irrespective of the magnitude. Effective normal stress of 1 MPa may be feasible where the fluid pressure is high. Under this condition, segregation takes only the slip displacement of 10 cm and the duration of 0.1 s. This may be satisfied in *M*_*w*_ > 7 earthquakes.

On the other hand, a critical comparison of the model with natural or experimentally sheared gouge is needed. Here we discuss two important points regarding the relevance of the present model to fault gouges in natural faults. The first important point is the grain-size distribution. Here we adopt a nearly bidisperse system, but natural fault gouges have power-law size distribution: smaller grains are more numerous than larger ones. Thus, it is not straightforward to apply the result of present simulation to natural faults. However, if the migration velocity just increases with the grain diameter, we can expect a similar segregation behaviour in granular systems with power-law size distribution. Thus, the systematic dependence of the migration velocity on the grain diameter is an important future task. For instance, an interesting result has been given by Golick & Daniels [30]. Based on such studies, one can gain more insight on the feasibility of grain-size segregation in natural fault gouges.

The second important point is the effect of pore fluid. Here, we neglect a pore fluid because the effect of fluid may not be very significant in a system of closely packed grains, where the inter-grain force is dominant over the fluid–grain interactions. However, one could still argue that the fluid might affect the segregation tendency. For instance, the drag force between the grains and the fluid reduces the grain inertia. As the dragging coefficient increases with the grain diameter, the dragging force may lead to a size-dependent migration velocities that is different from that of the dry case. It should be tested by massive numerical simulations in which the grain–fluid interactions are properly modelled.

## Data accessibility

This article has no additional data.
